# Phase-transition materials derived photonic metamaterials for passively dynamic solar thermal and coldness harvesting

**DOI:** 10.1016/j.heliyon.2024.e23986

**Published:** 2024-01-08

**Authors:** Hengliang Wu, Dan Shang, Huan Zhang, Lifeng Zhi, Shaolong Sun, Shiming Cui, Chaoqun Yan

**Affiliations:** a Shanghai Marine Diesel Engine Research Institute, Minhang, Shanghai, 201108 China; b Forth military representative office in Shanghai, Minhang, Shanghai, 201108 China

**Keywords:** Solar thermal conversion, Photonic manipulation, Radiative cooling, Phase transition, Vanadium dioxide

## Abstract

The rising need for energy to actively heat and cool human-made structures is contributing to the growing energy crisis and intensifying global warming. Consequently, there's a pressing need for a sustainable approach to temperature management that minimizes energy consumption and carbon emissions. The substantial temperature differences between the Sun (approximately 5800 K), Earth (around 300 K), and outer space (about 3 K) offer a unique opportunity for passive thermal regulation on a global scale. Recent research indicates the possibility of addressing this issue through various low-carbon, passive technologies such as solar heating and radiative cooling. However, their practical application is often limited to certain seasons and climatic regions due to their static and single-function nature in managing temperature. In this context, we introduce a concept of phase-change metamaterials that provide passive, dynamic, and adjustable radiative thermal control, suitable for widespread engineering applications. Our designed metafilm comprises a Polydimethylsiloxane (PDMS) layer infused with vanadium dioxide (VO_2_) nanoparticles, backed by a layer of broadband-reflective silver (Ag). This metafilm exhibits a self-adjusting solar absorptance, shifting from 0.96 to 0.25 at a pivotal temperature while maintaining a nearly constant thermal emittance. We can finely tune the metafilm's optical characteristics by altering the VO_2_ nanoparticle concentration and PDMS layer thickness. To demonstrate its efficacy in solar thermal management and radiative cooling, we simulate its temperature behavior under various weather conditions.

## 1 Introduction

Active thermal regulation in buildings, responsible for about 20% of worldwide electricity consumption, creates a significant demand for energy-efficient solutions for maintaining comfortable indoor temperatures [1], [2], [3]. Traditional compressor-based systems not only consume substantial energy but also contribute to greenhouse gas emissions, a factor in the increasing occurrence of extreme weather events [4], [5]. The Sun, with its approximate temperature of 5800 K, plays a dual role: it provides essential warmth to the Earth (around 290 K) during colder months, while also contributing to severe heatwaves in warmer periods [6], [7], [8]. Conversely, the vast coldness of outer space (about 3 K) offers a natural dissipative path for Earth's heat, especially through the atmospheric transparency in the 8 to 13 μm range [9], [10], [11]. This natural radiative exchange between the Sun, Earth, and outer space presents a viable alternative to conventional heating and cooling methods [6], [12]. For efficient solar heating, surfaces designed for photothermal conversion should have high solar absorptance (*α*) in the 0.3 to 2.5 μm solar wavelength range, crucial for solar energy capture. At the same time, all objects naturally emit infrared thermal radiation to their cooler surroundings [13], [14]. However, when these photothermal materials absorb solar energy, their efficiency is compromised by spontaneous thermal re-emission, attributed to high thermal emittance [15], [16], [17], [18]. Thus, engineering a surface with both high *α* and low thermal emittance is essential to enhance photothermal conversion efficiency and achieve higher stagnation temperatures [19]. The process of passive radiative cooling, which involves the Earth dissipating heat into the deep cold space through the transparent atmospheric window, is facilitated by high thermal emittance materials over this wavelength range [20]. Demonstrations of subambient nocturnal radiative cooling have been achieved using materials like polyvinyl-fluoride polymer film (TEDLAR), polyvinylidene difluoride composites, TiO_2_ paints, and SiO films [21], [22], [23], [24], [25]. To optimize daytime radiative cooling, a surface must be engineered to reflect solar energy while emitting infrared radiation [26], [27], [28]. An ideal radiative cooler would thus display high solar reflectance alongside high thermal emittance, whereas an optimal solar absorber would combine high *α* with low thermal emittance for efficient solar energy harvesting [29].

Leveraging recent progress in the field of radiative heat transfer and photonic control [26], [30], advancements in thermal photonic metamaterials are facilitating the evolution of passive solar heating and radiative cooling technologies. Spectrally selective solar absorbers, such as multi-layered metal-dielectric stacks [15], [31], cermets [32], [33], and plasmonic nanostructures [34], [35], have been validated with spectral manipulation achievements. The aforementioned surfaces through architecting structures of metals and dielectrics all involve challenging nanofabrication instruments [15], [32], [33], [36]. Subambient passive daytime radiative cooling has been realized by a broad array of artificial structures and materials, such as photonic polymers [8], [20], delignified cooling wood [37], nanophotonic structures [9], [10], and hierarchical polymer composites [38], [39], [40]. Even though spectrally selective solar heating and radiative cooling have been demonstrated, their optical features cannot vary from the cooling mode to the heating mode according to different application requirements, such as solar heating for building hot water supply or radiative cooling for the cooling demands to store cold water for daytime cooling needs. Therefore, a metasurface that can be switched between radiative cooling and solar heating that merge two functionalities into one structure, i.e., During the daytime, it can absorb solar energy and store it as hot water for human use, while during the nighttime, it can radiative cooling to cool water down and store the coldness for daytime building cooling demand. [41].

In recent investigations, vanadium dioxide (VO_2_), recognized for its role as a phase transition material, has emerged as a focal point in the exploration of dynamic optical property modulation for the purpose of radiative cooling. Ono et al. have put forth a concept for a self-adjusting radiative cooling device by amalgamating an infrared thermal regulator (VO_2_/magnesium fluoride (MgF_2_)/Tungsten (W)) with a selective filter employing an 11-layer stack composed of germanium (Ge)/MgF_2_ [42]. Kim et al., in their approach, harnessed a VO_2_/silicon (Si)/silver (Ag) stack as the thermal emissive component for radiative cooling, concurrently incorporating three-layered photonic crystals to regulate sunlight reflection [43]. Liu et al., on the other hand, introduced a nanoporous polyethylene (PE) film to reflect sunlight and diminish convection heat transfer effectively. They additionally employed a 1D grating pattern of VO_2_ atop polydimethylsiloxane (PDMS) as a stretchable thermal emissive surface [44]. Notably, the inherent infrared transparency of VO_2_ contributes to proficient radiative heat dissipation. Furthermore, varying stretching ratios of PDMS correspond to distinct stagnation temperatures due to their unique thermal emittance characteristics. Taylor et al. achieved adjustable thermal emittance through the use of a metafilm comprising a Si spacer, VO_2_ film, and an aluminum (Al) reflecting mirror [45]. Moreover, VO_2_ has been harnessed in metamaterials and metasurfaces to dynamically manipulate optical responses across both terahertz [46] and thermal wavelengths [47], [48].

While it is true that undoped VO_2_ exhibits a phase transition at around 68 ^∘^C, a value significantly above usual environmental temperatures, this challenge can be addressed by incorporating metal dopants like tungsten (W) and strontium (Sr). These dopants have the capability to significantly reduce the phase-transition temperature, bringing it down to approximately 25 ^∘^C, as demonstrated in previous research [49]. This property positions VO_2_ as an attractive candidate for the implementation of switchable radiative cooling systems. It's worth noting that the photonic structures mentioned earlier primarily focus on the manipulation of spectral characteristics within the mid-infrared wavelength range (5 − 25 μm). To address this, they often require the incorporation of solar reflectors or filters to manage solar heating effects. Furthermore, these structures rely on rigid silicon wafer surfaces, limiting their applicability to various curved surfaces in practical applications. Additionally, their spectral modulation capabilities tend to be less pronounced when it comes to solar wavelengths.

In our research, we suggest employing a metafilm composed of VO_2_ nanoparticles to achieve passive radiative thermal regulation, with a particular focus on solar wavelengths. This metafilm includes VO_2_ nanoparticles embedded in a PDMS layer, forming the foundational matrix, which is then layered over a slender Ag film. Due to its broad-spectrum reflectivity, we utilize the Ag thin film, covering both solar and infrared wavelengths (0.3 − 25 μm). The distinct molecular oscillations of PDMS endow it with selective emissivity in the infrared spectrum. This modulation in solar absorptance can be finely adjusted by varying the thickness of the VO_2_ nanoparticles embedded in the PDMS film and adjusting the volume fraction of VO_2_ nanoparticles. We have conducted simulations to illustrate the varying temperature control capabilities of the metafilm. Furthermore, we discuss a potentially scalable approach for industrial applications, highlighting the feasibility of scaling up the production process. The metafilm's ability to dynamically respond to temperature changes during both daytime and nighttime provides a versatile solution for addressing diverse building requirements, including hot water and cooling needs.

## 2 Fundamentals

### 2.1 Spectra calculation of the metafilm

In the context of our dynamic metafilm proposal, the structural design involves a multi-layered thin film architecture with the incorporation of nanoparticles. In situations where the structure comprises *N* layers, the generalized reflection coefficient at the interface between region *i* and region *i* + 1 can be described using the formula established by Chew and Lin [50].

(1) $${\overset{˜}{R}}_{i,i+1}^{\left(\mu \right)}=\frac{R_{i,i+1}^{\left(\mu \right)}+{\overset{˜}{R}}_{i+1,i+2}^{\left(\mu \right)}e^{2jk_{i+1,z}(d_{i+1}-d_{i})}}{1+R_{i,i+1}^{\left(\mu \right)}{\overset{˜}{R}}_{i+1,i+2}^{\left(\mu \right)}e^{2jk_{i+1,z}(d_{i+1}-d_{i})}}$$

In the described framework, we delve into the intricacies of the reflection coefficients within this multi-layered structure. Specifically, $R^{\left(\mu \right)}i,i+1$ represents the Fresnel reflection coefficient at the boundary separating layer *i* and i+1, while ${\overset{˜}{R}}^{\left(\mu \right)}i+1,i+2$ signifies the generalized reflection coefficient at the interface between layer i+1 and i+2. Here, *μ* takes on the values of either *s* or *p*, denoting the transverse electric or magnetic polarization, respectively. The variable $z=-d_{i}$ denotes the position of the *i*th interface, and $k_{i,z}=\sqrt{\epsilon_{i}(\omega )\omega^{2}/c^{2}-k_{\rho }^{2}}$ represents the normal *z*-component of the wave vector in medium *i*. The relative permittivity of medium *i*, denoted as $\epsilon_{i}(\omega )$, varies with the angular frequency *ω*, while *c* stands for the speed of light and $k_{\rho }$ represents the in-plane wave vector. It's important to note that with ${\overset{˜}{R}}_{N,N+1}^{\left(\mu \right)}=0$, the equation provided offers a recursive approach to determine the reflection coefficients across all regions within the layered structure. The generalized transmission coefficient for this layered slab is defined by [50].

(2) $${\overset{˜}{T}}_{1,N}^{\left(\mu \right)}=\prod_{i=1}^{N-1}e^{jk_{iz}(d_{i}-d_{i-1})}S_{i,i+1}^{\left(\mu \right)}$$

The expression here provides the hemispherical emittance [51]

(3) $$\epsilon (\omega )=\frac{c^{2}}{\omega^{2}}\int_{0}^{\omega /c}dk_{\rho }k_{\rho }\sum_{\mu =s,p}(1-|{\overset{˜}{R}}_{h1}^{\left(\mu \right)}{|}^{2}-|{\overset{˜}{T}}_{h1}^{\left(\mu \right)}{|}^{2})$$

The coefficients for effective reflection and transmission, represented as $\overset{˜}{R}h^{\left(\mu \right)}$ and $\overset{˜}{T}h^{\left(\mu \right)}$ respectively, are dependent on polarization. Their specific values are calculable through the application of Eqs. (1) and (2).

The effective dielectric function of Mie-resonance metamaterials is expressed using the Clausius-Mossotti equation [52], [53].

(4) $$\epsilon_{eff}=\epsilon_{m}\left(\frac{R^{3}+2\alpha_{R}VF}{R^{3}-\alpha_{R}VF}\right)$$

$\epsilon_{m}$ represents the dielectric function of the matrix, while $\alpha_{R}$ denotes the electric dipole polarizability. Additionally, *R* and *VF* correspond to the radius and volume fraction of the nanoparticles, respectively. The utilization of Mie theory is crucial for incorporating the size effects of doped nanoparticles. The formulation of electric dipole polarizability can be established by integrating insights from both the Maxwell-Garnett equation and Mie theory [54].

(5) $$\alpha_{R}=\frac{3jc^{3}}{2\omega^{3}\epsilon_{m}^{3/2}}a_{1,R}$$

where $a_{1,R}$ is the first electric Mie coefficient given by

(6) $$a_{1,R}=\frac{\sqrt{\epsilon_{np}}\psi_{1}(x_{np})\psi_{1}^{\prime }(x_{m})-\sqrt{\epsilon_{m}}\psi_{1}(x_{m})\psi_{1}^{\prime }(x_{np})}{\sqrt{\epsilon_{np}}\psi_{1}(x_{np})\xi_{1}^{\prime }(x_{m})-\sqrt{\epsilon_{m}}\xi_{1}(x_{m})\psi_{1}^{\prime }(x_{np})}$$

The Riccati-Bessel functions are represented by $\psi_{1}$ and $\xi_{1}$, which are further defined as $\psi_{1}(x)=xj_{1}(x)$ and $\xi_{1}(x)=xh_{1}^{\left(1\right)}(x)$. Here, the functions $j_{1}$ and $h_{1}^{\left(1\right)}$ denote the first-order spherical Bessel function and the first-kind spherical Hankel function, respectively. The symbol ‘′’ signifies the first derivative. The size parameters for the matrix and nanoparticles are given by $x_{m}=\omega r\sqrt{\epsilon_{m}}/c$ and $x_{np}=\omega r\sqrt{\epsilon_{np}}/c$, respectively. The dielectric function of the nanoparticles is represented by $\epsilon_{np}$. The dielectric functions for Ag and PE have been sourced from ref. [55] and ref. [56], respectively. For insulating VO2, which is anisotropic, the dielectric function $\epsilon_{O}$ applies in the ordinary mode perpendicular to the optical axis within the x−y plane, while $\epsilon_{E}$ pertains to the extraordinary mode along the optical axis. By leveraging the classical oscillator formula, $\epsilon (\omega )=\epsilon_{\infty }+\sum_{i=1}^{N}\frac{S_{i}\omega_{i}^{2}}{\omega_{i}^{2}-j\gamma_{i}\omega -\omega^{2}}$, both $\epsilon_{O}$ and $\epsilon_{E}$ can be deduced. The high-frequency constant $\epsilon_{\infty }$, phonon frequency $\omega_{i}$, scattering rate $\gamma_{i}$, and oscillator strength $S_{i}$ values are cited from ref. [57]. When in the metallic state, VO2 exhibits isotropy, and its dielectric function is defined using the Drude model [57] as $\epsilon (\omega )=\frac{-\omega p^{2}\epsilon_{\infty }}{\omega^{2}-j\omega \Gamma }$. By solving Eqs. (3)-(5), spectra of different metastructures can be calculated.

### 2.2 Energy balance analysis

To assess the thermal performance of the metafilm, we conducted simulations to establish thermal equilibrium using the following equation: [42], [58]:

(7) $$Q_{\text{total}}\left(T_{\text{metafilm}},T_{\text{amb}}\right)=Q_{\text{sun}}\left(T_{\text{metafilm}}\right)+Q_{\text{amb}}\left(T_{\text{amb}}\right)-Q_{\text{re-emit}}\left(T_{\text{metafilm}}\right)$$

In this context, the rear side of the metafilm is thermally shielded, meaning no thermal burden is associated with our suggested structures. This is because varying thermal loads lead to different thermal reactions. Energy exchange takes place between the metafilm, surrounding air, the Sun, and outer space. In this setup, $Q_{sun}$ denotes the heat gain from the sun, $Q_{amb}$ signifies the incoming thermal radiation from the atmosphere, $Q_{re-emit}$ indicates the metafilm's reemitted heat flux and $Q_{total}$ is the metafilm's overall heat flux.

The solar heating gain, $Q_{sun}(T_{metafilm})$, is defined by:

(8) $$Q_{\text{sun}}\left(T_{\text{metafilm}}\right)=A\cdot CF\int_{0}^{\infty }d\lambda I_{\mathrm{AM}1.5}(\lambda )\alpha \left(\lambda ,\theta_{\text{sun}},T_{\text{metafilm}}\right)$$

In this context, *A* represents the surface area of the metafilm. $\alpha \left(\lambda ,\theta_{\text{sun}},\right)$ $T_{\mathrm{metafilm}})$ defines the solar absorptance, which depends on temperature, wavelength, and angle. However, in subsequent sections, it's shown that its solar absorptance is independent of angle and temperature. Consequently, it's justifiable to use the solar absorptance determined at an incidence angle of zero for calculations.

The thermal power from the atmosphere in the form of radiation heat transfer, $Q_{sun}(T_{metafilm})$, can be expressed:

(9) $$Q_{\mathrm{amb}}\left(T_{\mathrm{amb}}\right)=A\int_{0}^{\infty }d\lambda I_{\mathrm{BB}}\left(T_{\mathrm{amb}},\lambda \right)\alpha \left(\lambda ,\theta ,\phi ,T_{\mathrm{metafilm}}\right)\epsilon (\lambda ,\theta ,\phi )$$

Here, $I_{\mathrm{BB}}\left(T_{\mathrm{amb}},\lambda \right)=2hc^{5}\lambda^{-5}\exp{\left(hc/\lambda k_{B}T_{amb}-1\right)}^{-1}$ denotes the thermal irradiance of a blackbody in relation to its temperature. In this equation, *h* is the Planck constant, while $k_{B}$ is the Boltzmann constant. The expression $\alpha \left(\lambda ,\theta ,\phi ,T_{\mathrm{metafilm}}\right)=\frac{1}{\pi }\int_{0}^{2\pi }d\phi \int_{0}^{\pi /2}\epsilon_{\lambda }\cos\theta \sin\theta d\theta$ represents the solar absorptance, which depends on temperature as detailed in [13]. However, based on the thermal stability results presented in the original study, this parameter is assumed to be temperature-independent. The air's emittance, ϵ(λ,θ,ϕ), is described as 1−t(λ,θ,ϕ). Here, t(λ,θ,ϕ) is the atmospheric optical transmittance derived from MODTRAN 4, as referenced in [59].

The heat flux spontaneously reemitted by the metafilm is calculated as follows:

(10) $$Q_{\text{re-emit }}\left(T_{\mathrm{metafilm}}\right)=A\int_{0}^{\infty }d\lambda I_{\mathrm{BB}}\left(T_{\mathrm{metafilm}},\lambda \right)\epsilon \left(\lambda ,\theta ,\phi ,T_{\mathrm{metafilm}}\right)$$

The metafilm's emittance, represented by $\epsilon \left(\lambda ,\theta ,\phi ,T_{\mathrm{metafilm}}\right)$, matches its absorptance $\alpha \left(\lambda ,\theta ,\phi ,T_{\mathrm{metafilm}}\right)$. This equivalence is a consequence of Kirchhoff's law of thermal radiation, as referenced in [60].

To determine the temperature response, the following formula can be used:

(11) $$C_{\mathrm{metafilm}}\frac{dT}{dt}=Q_{\mathrm{total}}\left(T_{\mathrm{metafilm}},T_{\mathrm{amb}}\right)$$

The metafilm is around 20 μm thick. Its heat capacitance, $C_{metafilm}$, is considered here to equal the heat capacitance of 20 μm PDMS film without considering the contribution of VO_2_ nanoparticles since its volume fraction is too low. Temperature response was simulated by solving Eqs. (7)-(11).

## 3 Result and discussion

### 3.1 The concept of passive dynamic temperature response by phase-transition metamaterials

**Figure 1:**
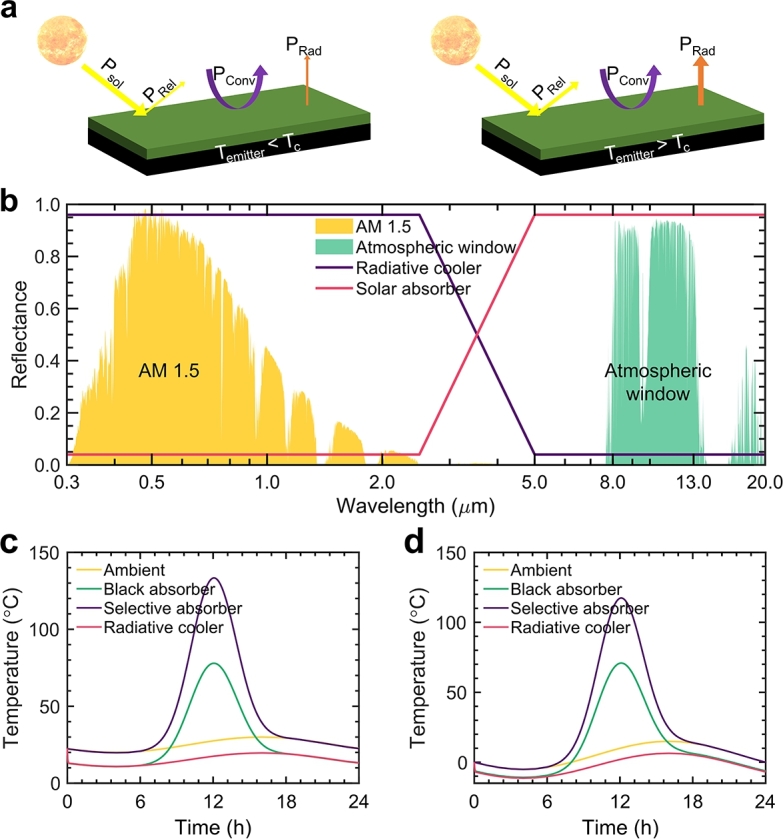
**Concept of switch between solar heating and radiative cooling by photonic metamaterials.** (a) Schematic illustrating the energy analysis of radiative cooling and solar heating. (b) The reflectance spectra of the solar absorber and radiative cooler for the heating and cooling modes, respectively. It displays the normalized stand solar irradiance spectrum and the transmittance spectrum of the atmospheric window. (c) Temperature variations of different substances: black absorber (green, approaching unity emittance over broad wavelengths from 0.3 μm to 25 μm), selective solar absorber (purple), and radiative cooler (red) compared with that of the ambient on a typical summer (c) and winter days (d).

Most objects, such as soil, sand, water, tree, etc., show a high thermal emittance over the infrared wavelengths (2.5 − 25 μm), which overlaps the atmospheric window, explaining the formation of dewdrop during the clear night. Infrastructures and vehicles also have similar optical properties as those of the soil and sand. All of them lack spectral selectivity across solar and infrared thermal wavelengths, meaning their solar absorptance closely mirrors their thermal emittance. Moreover, their thermal emittance is fixed, and cannot be tuned according to a certain temperature. Therefore, this will cause an overheating problem during the summer because of its high solar absorptance and extra heating load during the winter due to its high thermal emittance. For radiative cooling application, a surface with high solar reflectance and thermal emittance is required to minimize solar heating and maximum heat dissipation effect for alleviating the cooling loads of infrastructures, while a surface with high solar absorptance and low thermal emittance is favored for solar harvesting and depressing radiative heat dissipation during the cold winter. Another key point is the switchable property of these two different scenarios at a critical temperature, which can help alleviate the energy consumption of buildings. Fig. 1a depicts the energy transfer of a surface capable of dynamic optical responses varying with temperature. The modulation of convection heat transfer, not achievable solely through adjusting surface optical properties, won't be the focus of this study. Fig. 1b presents the reflectance spectra for the ideal radiative cooler and the solar absorber. Given the surface's opaqueness and thus a zero transmittance, reflectance is defined as 1 minus emittance. Figs. 1c and 1d clarify the temperature behaviors of the black absorber, the selective absorber, and the radiative cooler during two characteristic seasons. The maximum solar irradiance is designated at 900 W m^−2^ during midday, described by a Gaussian distribution, highlighting intensity variations from 6:00 AM to 6:00 PM. Ambient temperature variation is modeled by $T_{amb}(t)=T_{amb}^{avg}+\Delta T_{amb}sin[2\pi (t(h)-11)/24]$, with settings of $T_{amb}^{avg}$ = 25 ^∘^C, $\Delta T_{amb}$ = 5 ^∘^C for Fig. 1c, and $T_{amb}^{avg}$ = 5 ^∘^C, $\Delta T_{amb}$ = 10 ^∘^C for Fig. 1d. As demonstrated in Fig. 1c, the black absorber's temperature intriguingly dips below the ambient, attributed to its pronounced thermal emittance across the atmospheric window. Accounting for conduction, convection, and radiative transfers, an overall positive cooling power enables the object's temperature to fall beneath the ambient. Conversely, the selective solar absorber remains close to the ambient temperature, its subdued thermal emittance signifying minimized radiative heat dispersion. During daylight, both the black and selective solar absorbers attain temperatures substantially exceeding the ambient. The selective absorber's peak (133 ^∘^C) overshadows the black absorber's (78 ^∘^C), the former's minimal thermal emittance curbing radiative heat flow. Given its high solar reflectivity and thermal emittance, the radiative cooler retains temperatures below ambient both day and night. For a typical winter day (Fig. 1d), the black absorber faces the challenge of nighttime subambient temperatures, whereas the ever-subambient radiative cooler augments heating energy demands. The selective absorber mirrors ambient nighttime temperatures and accentuates solar heating for structures due to its heightened daytime temperature response. The radiative cooler proves exemplary for summer, while the selective absorber shines in winter. Thus, surfaces that can dynamically toggle between the optics of selective absorbers and radiative coolers based on specific temperature thresholds promise a seamless transition between solar heating and radiative cooling.

### 3.2 The switchable optical response of VO_2_ at a critical temperature

**Figure 2:**
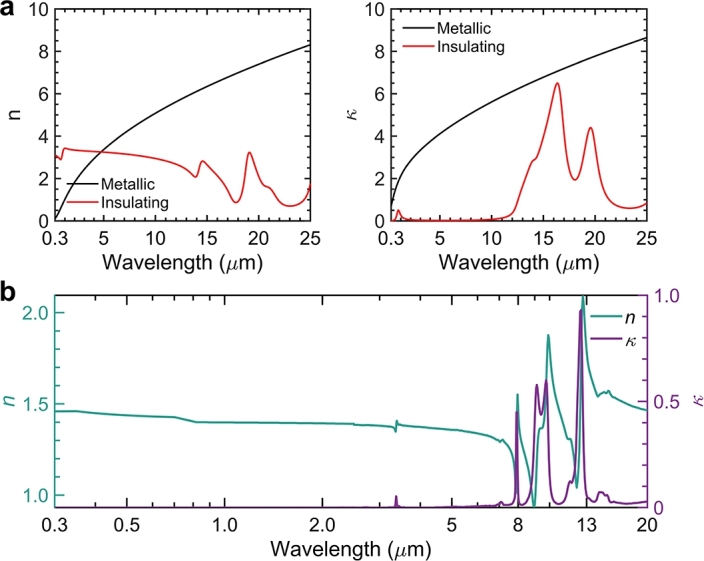
**Optical properties of VO**_2_**and PDMS.** (a) Complex refractive indices (*n* + *iκ*) of VO_2_ at different states. (b) Optical constant of PDMS over solar and infrared wavelengths.

Vanadium dioxide (VO_2_) is a phase-transition material known for its reversible shift from an insulating to a metallic state at a temperature of 68 ^∘^C. Below this critical temperature, VO_2_ functions as a semiconductor with a narrow gap, allowing it to be transparent to infrared light. However, exceeding this temperature transforms it into a metal-like state. This behavior is depicted through its varying complex refractive index, as shown in Fig. 2a. In the metallic phase of VO_2_, the refractive index (*n*) and extinction coefficient (*κ*) vary from 0 to 10, demonstrating an increase as the wavelengths extend, paralleling the characteristics of silver, as reported by Yang et al. (2015) [55]. This feature makes it an ideal candidate for infrared-reflective films aimed at mitigating infrared thermal radiation. In contrast, in its insulating phase, the *n* value hovers around 2, akin to dielectric materials, while the *κ* value is nearly negligible across most wavelengths, except in the 10 μm to 20 μm range. The wavelengths where *κ* is elevated coincide with the atmospheric window. VO_2_'s tunable radiative properties are also observable in its nanoparticle form [61] [62] [63]. The scalability of VO_2_ nanoparticles in industrial processes offers advantages over thin films, particularly in large-scale applications. Research has explored the hydrothermal method for producing VO_2_ nanoparticles, maintaining the phase-transition properties [64], [65] [66]. On the other hand, fabricating VO_2_ thin films typically involve vacuum deposition, a process that is more expensive and labor-intensive, as highlighted by Taylor et al. (2020) [45]. Further studies indicate that the complex refractive index of VO_2_ shifts within a specific temperature range around the transition temperature ($T_{c}$ ± ΔT). The dielectric function in this transition phase is described by the following equation:

(12) $$\epsilon \text{transition }=\arctan\left(\frac{T-Tc}{\Delta T}\times 10\right)\times \frac{\epsilon m-\epsilon i}{2\arctan10}+\frac{\epsilon m+\epsilon i}{2}$$

Here, $\epsilon_{m}$ and $\epsilon_{i}$ are used to denote the permittivities in the metallic and insulating phases of VO2, respectively. Introducing dopants such as molybdenum (Mo), tungsten (W), and strontium (Sr) into VO2 has been reported to reduce its phase transition temperature, as detailed by Dietrich et al. (2017) [49] and Liu et al. (2022) [44]. For example, research by Dietrich and team showed that doping with W notably lowers the phase transition temperature, evident in variants like V0.872Sr0.119W0.009O2 and V0.868Sr0.119W0.013O2. In our research, the phase transition temperature of the modified VO_2_ is set to *Tc* = 20 ^∘^C with a Δ*T* = 2 ^∘^C.

Fig. 2b displays the complex refractive indices of polydimethylsiloxane (PDMS) across a spectrum ranging from solar to infrared wavelengths, as detailed in studies by Zhou et al. (2019), Oyama et al. (2020), and Sun et al. (2016) [26], [67], [68]. PDMS exhibits remarkable transparency across solar wavelengths, akin to air, due to its minimal extinction coefficient spanning from 0.3 μm to 2.5 μm. This transparency is interrupted by notable peaks in the extinction coefficient (*κ*) within the infrared thermal wavelength range of 5 μm to 20 μm. These peaks arise from the molecular vibrations in the functional groups of PDMS, such as the symmetric CH_3_ deformation in Si-CH_3_ and the asymmetric Si-O-Si and Si-C stretching vibrations in Si-CH_3_, as indicated by Atta et al. (2022) [69]. These absorption peaks contribute to PDMS's effectiveness as a thermal emitter in the atmospheric window. However, manipulating the spectrum across such a broad range of wavelengths, from solar to infrared, presents challenges. This is particularly evident with VO_2_ nanoparticles, which display a grey hue and exhibit high absorption in solar wavelengths when present in large volumes. Conversely, reducing their volume fraction improves spectral manipulation capabilities in the solar range but results in diminished thermal emittance at infrared wavelengths.

### 3.3 Switchable solar absorptance of proposed metafilm

**Figure 3:**
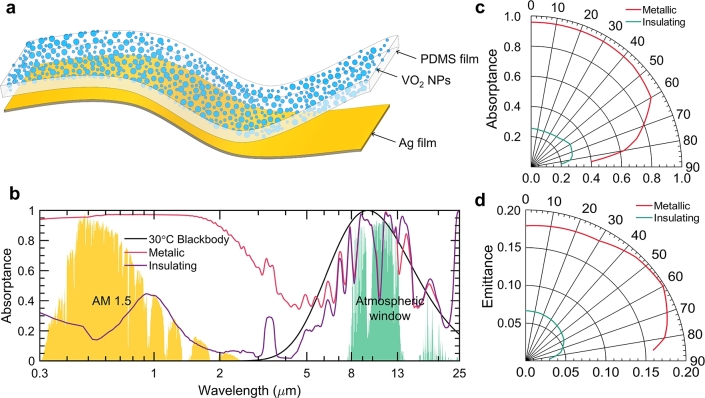
**Schematic and optical properties of the dynamic metafilm.** (a) Schematic displaying the structure of the metafilm. (b) Illustrations display the absorptance spectra of the metafilm in its metallic and insulating states, compared with the AM 1.5 standard spectrum and atmospheric transparency window. (c, d) The diagrams show the total solar absorptance (c) and thermal emission (d) of the metafilm in both its insulating (red) and metallic (green) phases at varying incident angles, demonstrating the metafilm's stable optical characteristics from 0^∘^ to 60^∘^.

Leveraging the distinct properties of PDMS film, such as its transparency over solar wavelengths, flexibility, and effective thermal emission in the atmospheric window, a scalable metafilm design has been conceptualized to enable a passively dynamic thermal response. This is achieved by adjusting solar absorptance in the solar wavelength spectrum through the phase-transition characteristics of VO_2_ nanoparticles, as illustrated in Fig. 3a. The metafilm is composed of 10 nm diameter VO_2_ nanoparticles, constituting a 0.7% volume fraction, embedded in a 10 μm thick PDMS film. The underside of the PDMS layer is coated with a 200 nm thick Ag film, which is opaque across both solar and infrared wavelengths. Consequently, the absorptance is equivalent to 1 minus the reflectance, as the emittance matches absorptance in thermally equilibrated objects.

Given the relatively small size of VO_2_ nanoparticles compared to the thickness of the PDMS film and their diluted concentration, Mie-theory is applicable for calculating the properties of the metafilm. The transparency of PDMS in solar wavelengths allows VO_2_ nanoparticles to demonstrate their optical characteristics and simultaneously acts as a thermal emitter within the atmospheric window. Fig. 3b presents the absorptance spectra of the metafilm in both its metallic and insulating states, contrasted with the normalized spectra of the standard AM 1.5 solar spectrum, the atmospheric window, and the 30 ^∘^C blackbody irradiation spectrum. The metafilm exhibits a solar absorptance exceeding 0.95, similar to a black absorber, in its metallic state, and maintains high thermal emittance due to the PDMS film's properties within the atmospheric window. In this state, VO_2_ nanoparticles mimic the behavior of metal nanoparticles like Cu, Au, and Ag, which possess localized surface plasmonic resonance for high solar absorptance [70]. Additionally, the random arrangement of VO_2_ nanoparticles enhances light trapping through scattering efficiency and multiple internal reflections and absorptions in the metafilm, achieving a solar absorptance of 0.95 despite their low volume fraction.

In its insulating state, VO_2_ behaves akin to a dielectric with low *κ* value in solar wavelengths, yet exhibits absorption peaks in thermal infrared wavelengths. The metafilm's solar absorptance drops to 0.25 in this state, as both the PDMS and insulating VO_2_ nanoparticles are transparent to solar wavelengths, and the Ag film reflects sunlight. The thermal emittance of the PDMS film is slightly increased by the sparse VO_2_ nanoparticles, particularly around 13 μm. Figs. 3c and d demonstrate the metafilm's consistent solar absorptance and thermal emittance in both metallic and insulating states, independent of angle. The innovative metafilm consistently exhibits constant solar absorptance ranging from 0^∘^ to 60^∘^ in all operational modes, a characteristic ascribed to the omnidirectional photonic arrangement of the VO_2_ nanoparticles embedded in the PDMS matrix. This feature effectively eliminates the necessity for sun-tracking mechanisms in solar energy harvesting. In the spectrum of mid-infrared frequencies, the metafilm demonstrates a universally even thermal emissivity, approximately 0.69, thereby augmenting radiative cooling efficiency. In its insulating phase, the metafilm sustains a consistent solar absorptance of approximately 0.25, diminishing solar heat intake from multiple angles. Despite its lack of reduced thermal emissivity in the atmospheric window while in its conductive state, hence not achieving spectral selectivity, this metafilm architecture signifies a notable progression in the adaptive control of solar absorptance through the use of phase-change materials.

### 3.4 Tunable thermal perfromance

**Figure 4:**
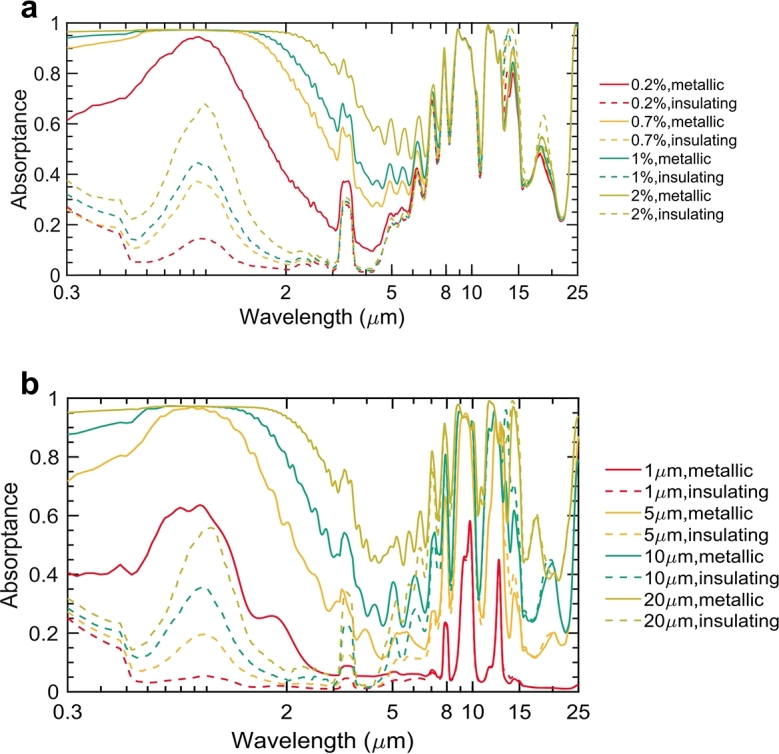
**Tunable optical response of the metafilm.** (a) The absorptance profile of the metafilm varies with different volume fractions of VO_2_ nanoparticles, maintaining a consistent PDMS film thickness of 10 μm. (b) The spectral absorptance characteristics of the metafilm are presented for varying thicknesses of the PDMS layer while keeping the VO_2_ nanoparticle volume fraction constant at 0.7%.

The spectra of our proposed metafilm, which are crucial for achieving different stagnation temperatures for various applications, are subject to tunability, as explored in Fig. 4a, b. The metafilm's spectral characteristics are influenced by alterations in its structural parameters, particularly the thickness of the PDMS film and the volume fraction of VO_2_ nanoparticles. While the thickness of the Ag film is also important for reflecting a broad range of wavelengths from solar to infrared, minor changes in the diameter of the VO_2_ nanoparticles do not significantly affect the absorptance spectrum.

By modulating the volume fraction of VO_2_ nanoparticles from 0.2% to 2%, while keeping the PDMS film thickness fixed at 10 μm, there is a marked enhancement for the values of solar absorptance. In the metallic phase, the absorptance increases from 0.7 to 0.96, whereas in the insulating phase, it advances from 0.12 to 0.43. Its thermal emittance remains comparatively uniform across the atmospheric window, a consequence of the relatively low VO_2_ nanoparticle volume fraction. Considering the trade-off between spectral adjustment efficacy and the expense of VO_2_ nanoparticles, a volume fraction of 0.7% is identified as optimal for substantial alteration in solar absorptance.

Fig. 4b highlights the spectral tunability of the metafilm as the PDMS film thickness varies from 1 μm to 20 μm, with the VO_2_ volume fraction held steady at 0.7%. This thickness variation leads to changes in both solar absorptance and thermal emittance. A thicker metafilm enhances both these properties, providing broader applicability in engineering projects that require specific target temperatures. This tunable solar absorptance feature of the metafilm opens up new possibilities for its application in diverse temperature-regulated environments.

**Figure 5:**
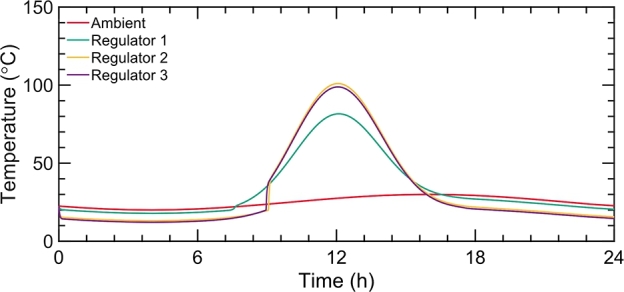
(a) The temperature behavior of three distinct structures, namely a black absorber, a selective solar absorber, and a thermal regulator, is analyzed under summer weather conditions.

Fig. 5 illustrates the adjustable stagnation temperature characteristics of the metafilm. The metafilm configurations, labeled as Regulators 1, 2, and 3, differ in their PDMS layer thicknesses of 1 μm, 5 μm, and 10 μm respectively, while maintaining an identical VO_2_ volume fraction. The thermal behavior of these metafilms is comparably consistent, reflecting their analogous spectral manipulation abilities as depicted in Fig. 4b. As the PDMS layer's thickness increases, a rise in temperature is noted during night hours, attributed to enhanced thermal emittance through the atmospheric window and improved heat dissipation capability. The peak temperatures recorded for these dynamic regulators are 81.6 ^∘^C, 101.2 ^∘^C, and 98.6 ^∘^C, correlating with their respective solar absorptance and thermal emittance at noon (as shown in Fig. 5a). The stagnation temperatures for Regulators 2 and 3 are markedly higher than Regulator 1, owing to their greater solar absorptance facilitated by the thicker PDMS layers containing VO_2_ nanoparticles. Despite Regulators 2 and 3 exhibiting higher thermal emittance than Regulator 1, the solar energy absorption surpasses the emitted thermal energy. Considering the stagnation temperature disparity between Regulators 2 and 3, although Regulator 3 has marginally superior solar absorptance compared to Regulator 2, its higher thermal emittance results in a slightly lower stagnation temperature.

The production methodology for the designed metafilm is notably scalable, aligning with engineering requirements. The VO_2_ nanoparticles are efficiently synthesized via the rapid hydrothermal technique, a method proven effective for mass production [64], [65], [66]. Utilizing the blade coating technique, a standard in polymer film manufacturing, the metafilm can be produced on a large scale by applying a mixture of VO_2_ nanoparticles and PDMS solution [71], [72]. For the application of the Ag thin film, methods such as electroplating or roll-to-roll physical deposition are suitable. These unique features make it excellent for solar and coldness harvesting for buildings where both heating and cooling are needed. Unlike static radiative cooling or solar heating methods, which are limited to specific application scenarios, this dynamic technique can be deployed without mechanical or other stimulus for changing its optical properties. The proposed fabrication method is scalable for industrial-level production. However, it is important to consider that prolonged exposure to air may cause oxidation of the VO_2_ material, despite its encapsulation within the PDMS polymer matrix. To ensure long-term performance, it is recommended to incorporate antioxidants into the system to extend the lifetime of both PDMS and VO_2_. Additionally, when applying this dynamic tunable structure in real-life scenarios, it is crucial to assess the UV-resistance capability of the PDMS film and take necessary measures to mitigate any potential degradation.

## 4 Conclusion

In summary, we have developed a temperature-regulating metafilm based on phase-transition materials. This metafilm demonstrates a capacity to modulate temperature near ambient levels, owing to its solar absorptance that dynamically responds to a critical temperature. The optical transition near this critical temperature allows the metafilm to modulate solar absorptance across solar wavelengths. It functions effectively as a solar absorber for energy harvesting in its metallic state. Conversely, its reduced solar absorptance coupled with high thermal emittance in the insulating state makes it an efficient radiative cooler. The flexible PDMS film provides a supportive matrix for embedding VO_2_ nanoparticles. However, due to the nanoparticles' low volume fraction, minimal photonic manipulation is observed at thermal infrared wavelengths in this structure. The spectral tunability of the metafilm enables a broader range of applications for various temperature control scenarios. This innovative metafilm is particularly suitable for integrating into building envelopes, aiding in cooling and heating functions. During the daytime, it serves as a solar absorber for storing hot water while acting as a radiative cooler for storing coolness in the nighttime. Although its high thermal emittance over atmospheric windows cannot provide thermal retention functionality for the cold nighttime in the winter, the cooling and heating mode for the metafilm shows the potential for energy-saving infrastructure applications.

## CRediT authorship contribution statement

**Hengliang Wu:** Writing – original draft, Conceptualization. **Dan Shang:** Visualization, Validation, Data curation. **Huan Zhang:** Validation, Software. **Lifeng Zhi:** Visualization, Validation. **Shaolong Sun:** Writing – review & editing, Visualization. **Shiming Cui:** Writing – review & editing, Writing – original draft. **Chaoqun Yan:** Writing – review & editing, Writing – original draft, Supervision, Conceptualization.

## Declaration of Competing Interest

The authors declare that they have no known competing financial interests or personal relationships that could have appeared to influence the work reported in this paper.

## Data Availability

No data was used for the research described in the article.
